# Surface Electromyography as a Method for Characterizing Mammogram Discomfort: Cross-Sectional Questionnaire Study of Procedural Stress

**DOI:** 10.2196/83971

**Published:** 2026-03-06

**Authors:** Krystyna Gielo-Perczak, Riley McNaboe, Hugo Posada-Quintero

**Affiliations:** 1 Department of Biomedical Engineering, College of Engineering, University of Connecticut, Arthur B Bronwell Bldg, 260 Glenbrook Road, Storrs, CT, 06269, United States, 1 860-486-0370, 1 860-486-2500

**Keywords:** mammogram, human factors, surface electromyography, procedural stress, discomfort

## Abstract

**Background:**

Mammograms are the most readily used procedures for early breast cancer detection but are notorious for the discomfort they induce in patients. This physiological strain has been validated by many questionnaire-based investigations, some of which indicate that it may discourage and deter women from potentially lifesaving health care. While informative, these subjective measures are highly variable and do not provide an objective perspective regarding the coordinated physiological and ergonomic response required for the procedure.

**Objective:**

A multimuscle surface electromyography (sEMG) methodology is proposed to better understand the machine-patient dynamics and potentially develop objective measures of mammogram-related pain and stress.

**Methods:**

Seven different muscle pairs on the neck, shoulders, and torso were identified as being critical in postural positioning during the industry-standard mammogram procedure. sEMG was recorded during 8 compressions across 2 mammogram simulations for 25 women wearing wireless devices. An illustrative map of muscle activity was created based on a comprehensive 10-metric sEMG analysis that compared baseline recordings with activated states during each compression.

**Results:**

The deltoid demonstrated the highest muscular activation across trials with an increase in the mean of the root mean square activity of up to 436%, while the trapezius upper fibers, infraspinatus, and teres major also showed significant increases in muscle activation, averaging 89% to 155% when compared with rest states. Across metrics, muscle activations were ipsilaterally correlated, with significant differences observed only when the breast was compressed on the same side as the muscle being measured. The serratus anterior and external oblique muscles showed minimal activation for any compression or positioning. No significant differences were found between curved and flat paddle designs. After the breast, patient-reported discomfort localized primarily to the shoulder and neck regions, corroborating the physiological measurements.

**Conclusions:**

The juxtaposition of muscle-specific activity against inactive muscles, along with reported discomfort values, demonstrates that the sEMG methodology accurately captures the patient-machine interaction dynamics with objective, quantifiable precision that complements subjective feedback. By providing real-time physiological data on muscular patterns and biomechanical responses throughout the procedure, this sEMG-based approach offers measurable metrics for evaluating proposed improvements to mammogram equipment design and protocols.

## Introduction

### Background

Charles Gross developed the first dedicated unit for obtaining radiographic images of breasts in 1965. Since then, mammogram machines and procedures have undergone continuous improvements in imaging quality, versatility, and accuracy [1]. However, many concerns have arisen, including high rates of false positives, radiation injury, overdiagnosis, and, most notably, procedure-induced pain [2,3]. This pain is commonly accepted by women who endure the taxing requirements of mammograms in hopes of better health [4]. Nonetheless, it is important to try to understand and address mammogram discomfort and related stress both out of compassion for patients and to encourage regular screening.

Understanding the manifestation of pain, both the physical toll it takes and the cognitive stress it induces, is inherently complex. While many researchers have used questionnaires and interviews to better understand mammogram-induced pain in women, a more complete methodology is required to fully examine all the variables that contribute to the discomfort of the procedure. A quantitative measure, such as that provided by surface electromyography (sEMG), can provide an objective perspective on the prevalence and intensity of mammogram-induced discomfort. sEMG is a noninvasive method using sensors on the skin to measure the change in voltage potential that occurs when a muscle is stimulated by the nervous system [5]. The primary waveform acquired is informative, as it reflects muscle activation resulting from the overall recruitment of fibers during contraction. sEMG also enables calculation of a wide range of secondary metrics, each of which can uniquely characterize different signal features as well as the physiological pathway that oversees their activity.

The depth of analysis that sEMG provides allows for a complex, multifaceted mapping of muscle activity, especially when multiple muscles are monitored simultaneously. Analysis of the signal and its related metrics has been used in a wide range of applications including, but not limited to, studies of comfort and ergonomics [6], emotion detection and regulation [7], and diagnostic techniques for muscular diseases [8]. Similarly, sEMG has been used in various approaches to identify and quantify pain and stress [9-11].

As a noninvasive method that can characterize a wide range of human states (eg, muscular demand and discomfort), sEMG is ideal for a comprehensive, quantitative biometric analysis of positioning and the resulting discomfort during mammograms. Such discomfort is realized when the technologist positions the patient into a forced and unnatural posture to ensure proper imaging. The patient stands with feet pointed at the machine and torso parallel to the imaging table while simultaneously rotating the head and grasping the side of the machine with a single arm. While holding this demanding position, patients must hold their breath and remain completely still. A detailed understanding of the muscular response of a patient during this machine-human positioning can provide greater context to fully understand the effect it has on the body. The breast-positioning procedure is extremely demanding on the patients’ muscles, ultimately inducing high physiological strain on the patient, a state that is further exacerbated by the intrinsic psychological stress associated with the screening itself.

Building upon our preliminary investigations presented in the studies by Gielo-Perczak et al [12,13], this study presents a comprehensive multimetric analysis of sEMG signals collected from 14 different muscles across 25 women during a traditional mammogram procedure. While previous work provided initial insights into a select range of muscles, the current investigation significantly expands both the scope and the depth of the analysis to evaluate the practicality and efficacy of using sEMG as a tool to understand the patient-machine dynamics and to quantify patient discomfort. The methodology seeks to understand the effects of different breast compressions, the required body positioning, and the overall physiological demand of the procedure. We analyzed the performance of 10 different sEMG metrics across each muscle throughout the baseline phases and stimulated (breast compression) phases. We then compared the difference in each metric response between the 2 phases to identify any significant trends resulting from the force of the paddles and the positioning required for the imaging.

### Previous Work

The application of sEMG to understand muscular distress or discomfort and the ergonomics of specific tasks or procedures has been widely explored. This work generally involves naturalistic observation whereby a muscle and an electromyography (EMG) metric are analyzed throughout a given task. Some groups expand upon this approach by including additional muscles or metrics but rarely both. By monitoring the signal during either real or simulated activities, sEMG has been used for diagnostic purposes as well as for targeting specific, localized task characteristics. This technique has helped to identify the physiological impact and strain of specific, observed tasks on participants, such as workers, patients, surgeons, or even musicians.

For example, Steinmetz et al [14] recorded sEMG from the sternocleidomastoid muscle of 2 groups of violinists—those with and those without neck pain. By analyzing the root mean square (RMS) of the signal, the research simultaneously observed the effect of violin playing on the muscle and examined how the presence or absence of pain could be captured with the signal. A similar, single-muscle approach was conducted by de Oliveira and Nadal [15], who analyzed the erector spinae of helicopter pilots to evaluate the effect of required in-flight posture and environmental vibrations on their back muscles. In addition to analyzing the RMS of the signal, they derived the median frequency to quantify muscle fatigue more specifically over time, allowing for a wider analysis. Harnessing a similar multimetric approach, Wijsman et al[16] and collaborators targeted the trapezius upper fibers during a cognitive task representative of work-related stress. They analyzed 9 different sEMG metrics including RMS, mean value of the RMS, median value of the RMS, peak value of the RMS, mean frequency of the signals-derived power spectra, median frequency of the signals-derived power spectra, and trends. Almost all the metrics indicated a significant difference when comparing the response between a given stressed and relaxed state, and some simultaneously exhibited differences between *types* of stressed states. The resulting multimetric visual gave a more profound interpretation as the frequency-based features were analyzed for muscle fatigue over the task period. RMS-based metrics were used to quantify the overall activation, while trends allowed for a synthesized report of RMS activity over time.

In each of these studies, the tasks required not only the targeted muscles but also many other muscles that were not a primary focus. While a multimetric approach like Wijsman’s [16] provides a more profound analysis of sEMG from a given muscle, a broader perspective is gained from evaluating entire task-relevant sections of the body. Bhardwaj et al [17] implemented such an approach in a study of truck driver fatigue. Bhardwaj’s team collected bilateral sEMG from 3 different posterior muscle groups (trapezius medial, latissimus dorsi medial, and erector spinae) as drivers operated both semitrucks and simulators. Although they implemented only 1 metric, the additional muscle data provided a more comprehensive view than if only a single muscle was selected. Both Luttmann et al [18] and Niu et al [19] analyzed multiple sEMG metrics to understand the ergonomics or effects of performing surgeries. Luttmann’s team used electrical activity (a metric indicative of smoothed and rectified sEMG) and median frequency to characterize sEMG from 3 different posterior muscles over a series of repeated surgeries. With this approach, they were ultimately able to quantify the muscular strain and fatigue. Niu and colleagues [19] observed surgeons’ arm muscles during laparoscopic and robotic surgeries to assess biomechanical stress. The analysis focused on integrated electromyography and mean frequency derived from 4 muscles located in the lower and upper arms. These 2 research teams, among others, demonstrate the value of a multimetric, multimuscle approach when implementing sEMG as a quantitative tool.

Often, a single muscle observation is sufficient to satisfy study objectives. However, when the study seeks a more complete illustration of muscular-related physiology (including factors such as posture, pain, fatigue, or stress), additional muscle observations augment the context for interpreting the results. Similarly, using multiple sEMG features in the analysis allows for more profound conclusions. The studies previously mentioned are summarized in Table 1.

**Table 1.** Previous studies.

Reference	Participants, n	EMG^a^ application	Methodology	Muscles analyzed	Metrics used	Comparison method
Wijsman et al [16] (2013)	30	Cognitive stress	Nine-features analysis of EMG to predict stressed state during a simulated cognitive stress test (VAS^b^ and PSS^c^)	Trapezius upper fibers	RMS^d^, gaps/minute, relative gap time, static/median/peak RMS, mean/median frequency, and trends	% RVC^e^
Steinmetz et al [14] (2016)	55	Task-related muscle function	One-feature analysis of sEMG^f^ from violinists with and without neck pain, to understand the impact of playing on pain of the muscle (VAS and NDI^g^)	Sternocleidomastoid	RMS	Δ *|*RMS*|*
Luttmann et al [18] (1998)	4	Muscular strain	Two-feature analysis of sEMG from urologists over repeated surgeries to analyze muscular strain and fatigue	Trapezius, deltoid, and erector spinae	EA, median frequency	% Maximum EA^h^ and Δ |MF|^i^
Bhardwaj et al [17] (2018)	25	Driver fatigue	One-feature analysis to compare efficacy of using EMG to analyze driver fatigue in simulators in comparison with real-life scenarios	Trapezius medial, latissimus dorsi medial, and erector spinae	Mean frequency	Coherency
de Oliveira and Nadal [15] (2004)	12	Occupational muscular fatigue	Two-feature analysis of EMG in comparison with MVC^j^ to evaluate the effect of posture and vibration on helicopter pilots’ back muscle fatigue while flying	Erector spinae	RMS, median frequency	% MVC
Niu et al [19] (2020)	7	Biomechanical stress	Two-feature analysis of surgeons’ lower and upper arms while conducting laparoscopic and robotic surgery to evaluate the difference in biomechanical stress to improve the surgery protocol or ergonomics	Flexor carpi radialis, brachioradialis, biceps brachii, and medial deltoid	iEMG^k^, mean frequency	% MVC

a EMG: electromyography.

b VAS: visual analog scale.

c PSS: Perceived Stress Questionnaire.

d RMS: root mean square.

e RVC: reference voluntary.

f sEMG: surface electromyography.

g NDI: Neck Disorder Index.

h EA: electrical activity.

i MF: median frequency.

j MVC: maximum voluntary contraction.

k iEMG: integrated electromyography.

## Methods

### Study Design

The proposed sEMG methodology to evaluate patient stress and discomfort during mammograms consists of a multimuscle, multimetric analysis. Researchers applied wearable sensors to 25 participants to simultaneously collect sEMG from 14 different relevant muscles during a simulated mammogram. The recordings collected during the imaging phase provide a comprehensive snapshot of muscular states during compressions. An in-depth analysis of 10 unique sEMG metrics provides important contextual information for the muscular mapping.

### Mammogram Positioning and Muscle Selection

The vertical, standing system is one of the most widely used imaging methods for mammograms. While slight variations can be seen between systems depending on the manufacturer, the overall patient-machine interaction remains consistent throughout the field. During the procedure, the patient stands with both feet facing toward the machine and torso pressed against the edge of an imaging table that protrudes from the device. The patient’s head is rotated significantly to the left or the right (depending on the breast being imaged), with the side of the face pressed against a rigid shield. The arm opposite to the breast being imaged simultaneously extends toward the machine.

Considering the rigid structures of the machine as well as the stationary placement of the feet, there are several muscles that can be evaluated to capture the restricted trunk torsion in conjunction with the head rotation and arm extension. The serratus anterior and external oblique can be observed to characterize relevant activity in the torso region. The deltoids along with the infraspinatus and teres major groups provide insight into the shoulder and upper arm during arm extension. The sternocleidomastoid and trapezius upper fibers together illustrate neck movements during rotation and extended flexion.

The left and right pairs of these 7 muscles (14 muscles total) were selected for observation in the proposed methodology. These muscles consisted of previously studied muscles [20] as well as a selection of new muscles that targeted areas not yet evaluated. It is important to note that none of the muscles identified are on or in direct contact with the breasts. The breasts themselves have no muscles and consist of lobules and ducts encapsulated by fatty adipose tissue. The proposed sEMG methodology focuses on muscle strain as a result of patient-machine positioning rather than the force applied to the breast itself.

### Instrumentation

**Figure 1.**
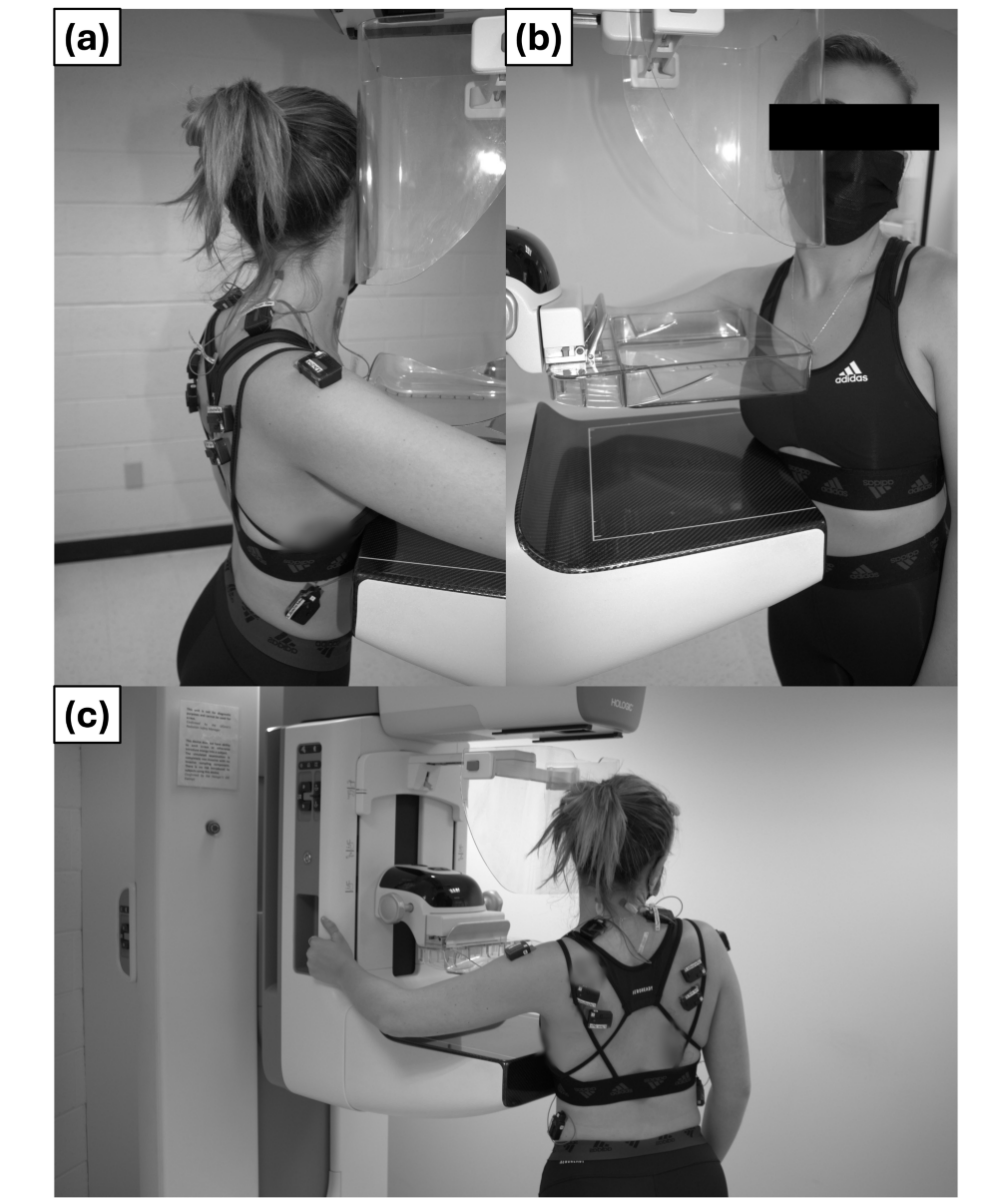
Example of human-machine positioning for compression and imaging (note: research staff member shown in images; participants were in a hospital gown for the duration of the study). (A) Lateral view of right craniocaudal compression position, (B) front view of right craniocaudal compression, and (C) rear view of left craniocaudal compression.

Surface EMG recordings were taken using wearable Trigno and Avanti sensors from Delsys, Inc. Four different sensor types were used including the Avanti, Avanti Duo, Trigno, and Trigno Mini. The type of sensor used was determined by the characteristics of the corresponding muscle and the restrictions presented by the procedure. The Avanti and Trigno sensors include embedded electrodes and are ideal for direct placement on muscles that exhibit limited movement during the imaging (placed parallel to the muscle fiber pattern on the center medial deltoid, lowest rib on the external abdominal oblique, center infraspinatus, and center teres major). The Mini and Duo systems use external reference electrodes connected by a wire to the measured electrodes, making them more suitable for smaller, isolated muscle areas that show more movement during the procedure (placed parallel to the muscle fiber pattern on the center trapezius upper fibers, center sternocleidomastoid, and the center band of the serratus anterior). Despite differences in electrode configuration, all 4 Delsys sensor models share identical sampling rate, bandwidth, and input range specifications, providing consistent EMG signal characteristics. To account for any minor variability intrinsic to sensor design or placement, all EMG signals were normalized prior to analysis to ensure valid comparisons across sensors and participants. Both the Avanti and Trigno sensor systems use default sampling defined by Delsys (1925.925 Hz). Figure 1A-C shows the localization of some of these sensors.

### Simulation

The mammogram procedure was simulated using the 3Dimensions Genius Mammography System from Hologic, Inc. This system (Figure 1) is commonly found in medical offices across the country and is similar to many other brands. Two different paddle designs—a flat, industry standard paddle and a newer Hologic SmartCurve Breast Stabilization Paddle—were used throughout the protocol to observe whether these design modifications produced any response differences [21]. A certified mammography technologist adjusted the height of the machine and positioned the breast for the simulated compressions to ensure proper handling of the patient and to provide a realistic simulation experience.

### Ethical Considerations

The University of Connecticut Institutional Review Board for human participant research approved all the study procedures (protocol H20-0146). Participants gave consent after reviewing the subject protocol.

All identifiable information collected from the participants was numerically coded and anonymized.

Compensation of US $100 was given to participants after completing the visit for the study.

### Protocol

The study enrolled 25 healthy female volunteers (aged 40‐67, median 50.8, SD 8 years; mean height 163.7, SD 7 cm; mean weight 78.0, SD 21 kg; and mean BMI 28.9, SD 6 kg/m^2^). The sample size was determined based on practical considerations such as recruitment feasibility and clinical workflow constraints, and it aligns with typical cohort sizes in similar procedural studies, which often include 10 to 20 participants [20]. While both men and women are susceptible to breast cancer, only biological female volunteers were selected for this study as breast cancer, which requires repetitive mammography screenings, is significantly more common in this population.

Participants were screened using a preliminary questionnaire that flagged allergies related to any of the study equipment and identified previous injuries or scars that could impact the procedure. Participants were required to have previously completed at least 1 standard mammogram procedure to provide accurate feedback. Additional self-reported information was collected, including previous mammogram visual analog scale (VAS) pain or stress scores from 1 to 10 and a reference level for physical activity derived from grip strength recordings [22].

Once screened, the participant changed into a hospital gown in a private room. The participant’s left and right deltoid, infraspinatus, teres major, external oblique, serratus anterior, trapezius upper fibers, and sternocleidomastoid were equipped with an array of wearable, wireless EMG sensors. Prior to the placement of any device, the skin at each electrode location was cleaned with 70% isopropyl alcohol. The placement and orientation of each sensor were kept constant for all participants to aid in signal analysis.

An initial EMG control recording (C1) was taken in a bright, large room (1341 square feet) during which the participant stood still in a relaxed position facing forward. This recording, as well as all subsequent EMG control recordings, lasted 2 minutes. The participant was then guided to the Hologic Mammography system, which was housed in a smaller, quiet, moderately lit room (171 square feet) with an ambient temperature ranging from 26 °C to 27 °C. Another control recording (C2) was taken in this smaller room before starting the simulated mammogram (Table 2).

**Figure 2.**
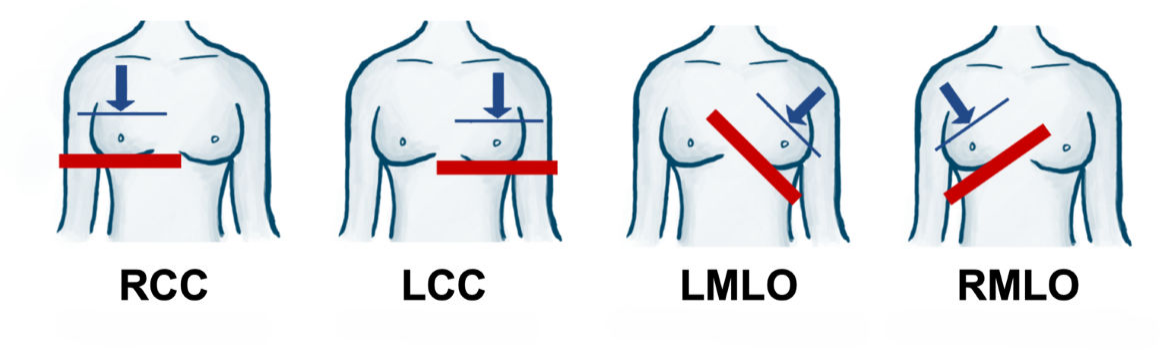
The 4 different positions of the breast for the simulated mammogram—typical for screening procedures. Blue line and arrow indicate paddle and direction of compressive force, respectively. Red line indicates imaging table on which the breast rests while being compressed. LCC: left craniocaudal; LMLO: left mediolateral oblique; RCC: right craniocaudal; RMLO: right mediolateral oblique. Adapted from Gielo-Perczak et al [12,13].

The team then initiated the mammogram simulation. The certified mammography technologist walked the participant through the procedure, which followed the same order of compressions currently used in the health care field. The compressions took the following order: (1) right craniocaudal (RCC) view (the right breast is compressed vertically), (2) left craniocaudal (LCC) view (the left breast is compressed vertically), (3) left mediolateral oblique (LMLO) view (the left breast is compressed laterally), and (4) right mediolateral oblique (RMLO) view (the right breast is compressed laterally). Figure 2 illustrates the 4 compressions. Both CC and MLO views require the participant to grasp the imaging unit for stability. In MLO views, the imaging table extends beneath the axilla, creating a larger body-machine contact surface and placing greater positional demands on the upper arm and shoulder girdle relative to the more vertically oriented CC positioning. Each of the 4 compressions (15 seconds each) was conducted twice, once using the flat traditional paddle (F_) and again with the curved SmartCurve Hologic Paddle (C_). The EMG recording was collected during the simulated imaging portion of the procedure when the breast was fully compressed, and the participant was in the restricted body position holding their breath. The compression force used was less than that which is typically applied in clinical settings. This was done to limit unnecessary participant pain since this work focuses on strain from patient-machine positioning rather than breast compression pain. No X-ray was emitted in this procedure. All other steps followed standard clinical practice. A third control recording (C3) was taken between the first and second sets of 4 compressions when the paddle was being changed. The entire protocol is summarized in Table 2.

**Table 2:** **.** Study protocol.

Compression event	Abbreviations	Duration
Control 1 in large room	C1	2 minutes
Control 2 in examination room	C2	2 minutes
Flat paddle compressions		
Right craniocaudal compression	F_RCC	15 seconds
Left craniocaudal compression	F_LCC	15 seconds
Left mediolateral oblique compression	F_LMLO	15 seconds
Right mediolateral oblique compression	F_RMLO	15 seconds
Control 3 in examination room	C3	2 minutes
Curved paddle compressions		
Right craniocaudal compression	C_RCC	15 seconds
Left craniocaudal compression	C_LCC	15 seconds
Left mediolateral oblique compression	C_LMLO	15 seconds
Right mediolateral oblique compression	C_RMLO	15 seconds

### EMG Signal Processing and Data Analysis

The sEMG processing and analysis was completed using MATLAB. The first and last 1.5 seconds were removed from the beginning and the end of each recording to remove fringe artifacts resulting from the misalignment with the start of the recording and the start of the imaging phase. The trimmed segment was then filtered with a fourth-order bandpass filter (20-500 Hz), which accounted for the variability in unique frequencies across the targeted muscles. Each segment for the flat paddle was normalized to the first control of the small room (C2), and the second set of recordings corresponding to the curved paddle was normalized to the second control of the small room (C3).

Ten different metrics were then derived from the cleaned signals to characterize each of the segments. The selection of these metrics was informed by prior literature in which similar measures have proven effective for assessing muscular pain, stress, fatigue, and strain in physically demanding contexts (Table 1). Five time-domain metrics were selected. These include the mean of the root mean square (meanRMS) waveform, standard deviation of the RMS (stdRMS) feature, slope sign change count, zero crossing count, and the waveform length (WFL). Five frequency domain metrics were selected to further analyze the EMG activity during each state of the protocol. These included the mean frequency (meanFreq), median frequency (medianFreq), signal spectral entropy, drop in power (DP), and spectral deformation (SpecDef). To determine these features, the power spectrum of each EMG signal was calculated using Welch’s periodogram method with 50% overlap and a 128-sample Blackman window on the filtered data. The average of each metric for a measurement window was used for analysis. Given the static nature of the participants during each measurement period and observed uniformity in sEMG data, limited variation in metrics was expected across each window.

### Data Analysis

Three interactions between stages of the protocol were identified as potentially illustrative comparisons. The first interaction illustrates the sEMG activity between the compressive state and its respective control. This finding highlights the sensitivity of the measure to illustrate the change from a relaxed to aroused state while also indicating overall muscle activity (eg, C_RCC vs C3). The second interaction revealed the sEMG activity between the same compressive state between the 2 different paddle types. This interaction would indicate any difference in muscle activity resulting from a change in the paddle form (eg, F_RCC vs C_RCC). The third interaction revealed the sEMG activity that occurred when the compression angle was changed for a given breast to identify any effect of angle on the signal (eg, F_RCC vs F_RMLO). Normality for each metric was assessed using the Kolmogorov-Smirnov test. Metrics were analyzed using paired *t* tests. Because each metric involved multiple comparisons across compressions, *P* values were adjusted using Bonferroni correction. Bonferroni was selected due to its conservative nature and its suitability for protecting against type 1 errors in settings with many planned comparisons.

## Results

**Figure 3.**
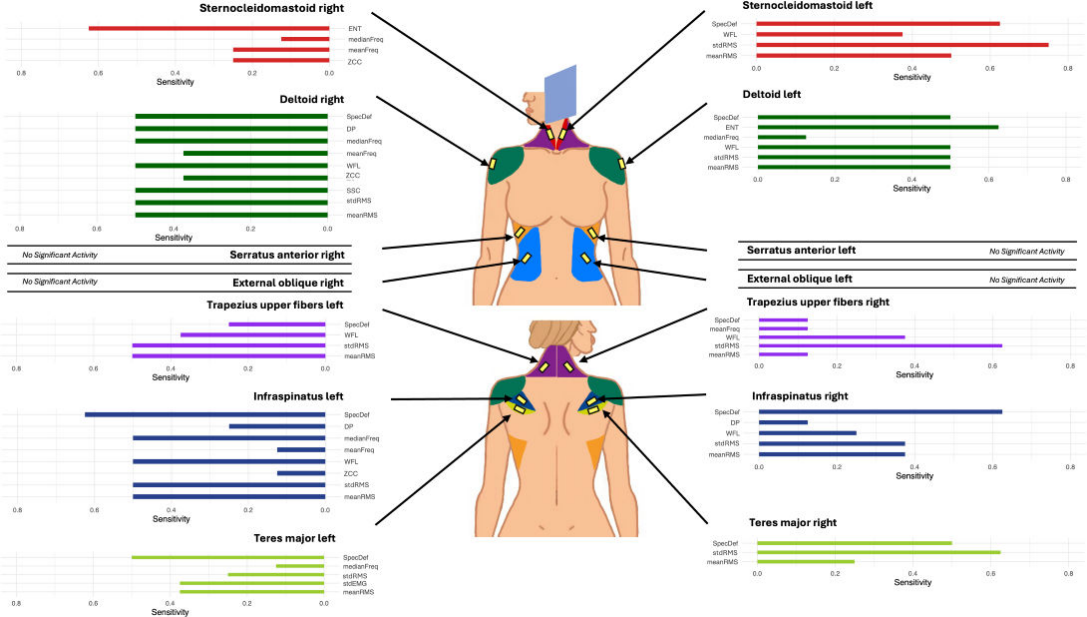
Percentage of compressions that showed a statistical difference between control and baseline for each muscle across all metrics. Displayed as a ratio out of the total number of compressions (n=8). Only metrics that showed significance for a given muscle are displayed. DP: drop in power; ENT: spectral entropy; meanFreq: mean frequency; meanRMS: mean of the root mean square; medianFreq: median frequency; SpecDef: spectral deformation; SSC: slope sign change count; stdRMS: standard deviation of the root mean square; WFL: waveform length; ZCC: zero crossing count.

The first interaction comparing activity during a compression to a control state was identified as the most informative with respect to the main goals of the proposed methodology. For this comparison, the metrics for a given muscle were evaluated across all 8 compression segments and the 2 small room controls C2 and C3. Statistical difference was noted according to the corrected Bonferroni values. This resulted in a total of 8 comparisons for a given metric and muscle combination. Figure 3 summarizes the percentage of compressions where this comparison was significant. The metrics listed for each muscle are those that detected a significant difference in sEMG activity at least once between relaxed and stimulated states for a given muscle.

The deltoid and infraspinatus muscles (left and right) demonstrated high relative sensitivity according to the frequency matrix when observing meanRMS, stdRMS, WFL, meanFreq, medianFreq, and SpecDef. The deltoid consistently showed differences in about 50% of cases. Similarly, the trapezius upper fibers and the teres major were also relatively sensitive to compressions when observing meanRMS, stdRMS, WFL, and SpecDef. The serratus anterior and the external oblique did not show any sensitivity to the compressions for any of the metrics explored. Regardless of the other muscles, meanRMS, stdRMS, WFL, mean and medianFreq, and SpecDef all indicated a change in sEMG between the 2 states. It is noted that meanRMS, stdRMS, and WFL are all somewhat correlated in addition to meanFreq and medianFreq. While each is slightly different (as they underscore various characteristics of the signal), the methodology of their derivation lends to shared trends, providing flexibility to researchers in metric selection when pursuing similar methods.

Those muscles that showed both frequent and significant responses across various metrics (all but the serratus and external oblique groups) are included in Table 3. The top 3 most frequently activated metrics in each muscle group are present for both paddle types. Any compression resulting in a significant change in comparison to the controls is noted with an asterisk and red font. Note that only C2 and C3 recordings are included in the table as they were the reference for the flat and curved paddle compression sets, respectively. No significant difference was found between the large room control C1 and C2 or between C1 and C3 in the examination room.

**Table 3.** Results for left and right muscle pairs across selected metrics. Most responsive metrics for each muscle pair. Values are presented as mean (SD).

	Control	Right craniocaudal	Left craniocaudal	Left mediolateral oblique	Right mediolateral oblique
**Sternocleidomastoid**					
Left					
meanRMS^a^ (mv)					
Flat	0.9481 (0.021)	1.569 (0.805)	**1.757 (0.861)** ^b^	**1.872 (1.18)** ^b^	1.399 (0.692)
Curved	0.9331 (0.0383)	1.547 (0.863)	**2.199 (1.12)** ^b^	**2.13 (1.39)** ^b^	1.429 (0.757)
stdRMS^c^ (mv)					
Flat	1 (1.052e-15)	**1.594 (0.707)** ^b^	**1.939 (0.91)** ^b^	**2.037 (1.26)** ^b^	1.703 (0.972)
Curved	1 (7.21e-16)	**1.883 (1.14)** ^b^	**2.432 (1.25)** ^b^	**2.204 (1.28)** ^b^	1.616 (0.896)
ENT^d^ (nu)					
Flat	0.7176 (0.016)	0.7088 (0.0138)	0.7071 (0.0125)	0.7116 (0.0204)	0.7115 (0.0169)
Curved	0.7019 (0.0307)	0.7024 (0.0258)	0.7098 (0.0129)	0.7103 (0.017)	0.7076 (0.0194)
Right					
meanRMS (mv)					
Flat	0.9634 (0.007)	1.701 (1.12)	1.097 (0.38)	1.055 (0.253)	1.807 (1.56)
Curved	0.959 (0.00943)	1.444 (0.789)	1.207 (0.49)	1.166 (0.37)	1.939 (1.48)
stdRMS (mv)					
Flat	1 (1.069e-15)	1.838 (1.21)	1.163 (0.385)	1.099 (0.258)	2.068 (1.86)
Curved	1 (6.86e-16)	1.434 (0.661)	1.434 (0.791)	1.278 (0.458)	2.211 (1.63)
ENT (nu)					
Flat	0.7277 (0.011)	**0.7152 (0.0118)** ^b^	0.7202 (0.00728)	0.7207 (0.00168)	**0.7084 (0.0172)** ^b^
Curved	0.7252 (0.00647)	**0.7054 (0.021)** ^b^	**0.7173 (0.00345)** ^b^	0.7192 (0.00556)	**0.708 (0.0181)** ^b^
**Trapezius upper fibers**					
Left					
stdRMS (mv)					
Flat	1 (7.96e-16)	**1.493 (0.447)** ^b^	**2.158 (1.26)** ^b^	**2.35 (1.32)** ^b^	**1.51 (0.513)** ^b^
Curved	1 (7.05e-16)	**1.883 (1.14)** ^b^	**2.432 (1.25)** ^b^	**2.204 (1.28)** ^b^	1.616 (0.896)
WFL^e^ (nu)					
Flat	6986 (893)	8927 (2450)	**15,360 (9420)** ^b^	**14,090 (6330)** ^b^	**9954 (3190)** ^b^
Curved	6255 (1140)	8257 (3340)	9558 (5250)	11,080 (6560)	10,960 (7160)
SpecDef^f^ (Ω/Ω^g^)					
Flat	2.001 (0.302)	1.874 (0.369)	**1.609 (0.411)** ^b^	**1.569 (0.415)** ^b^	1.761 (0.441)
Curved	2.039 (0.333)	1.884 (0.396)	1.732 (0.36)	1.805 (0.441)	1.848 (0.389)
Right					
stdRMS (mv)					
Flat	1 (1.09e-15)	**1.448 (0.537)** ^b^	1.393 (0.605)	**1.784 (1.01)** ^b^	**2.213 (1.49)** ^b^
Curved	1 (9.12e-16)	1.434 (0.661)	1.434 (0.791)	1.278 (0.458)	2.211 (1.63)
WFL (nu)					
Flat	6528.9 (1019.34)	**9596 (3550)** ^b^	**8996 (2590)** ^b^	**10,750 (5180)** ^b^	12,060 (7320)
Curved	6168 (1610)	8495 (4650)	9464 (6120)	8796 (5050)	11,530 (7670)
SpecDef (Ω/Ω)					
Flat	1.9636 (0.2187)	1.711 (0.419)	1.739 (0.31)	1.749 (0.456)	**1.569 (0.425)** ^b^
Curved	1.913 (0.257)	1.787 (0.337)	1.811 (0.319)	1.836 (0.327)	1.779 (0.476)
**Deltoid**					
Left					
meanRMS (mv)					
Flat	0.9655 (0.00909*)*	1.374 (0.588)	**4.814 (2.44)** ^b^	**5.287 (3.64)** ^b^	1.176 (0.416)
Curved	0.9518 (0.0199)	1.073 (0.28)	**4.195 (1.91)** ^b^	**4.996 (3.17)** ^b^	1.196 (0.517)
WFL (nu)					
Flat	7198 (898)	9563 (4460)	**32,880 (17,800)** ^b^	**33,420 (23,800)** ^b^	7874 (2980)
Curved	6368 (1250)	7076 (2130)	**27,310 (13,100)** ^b^	**36,760 (27,600)** ^b^	7723 (3140)
SpecDef (Ω/Ω)					
Flat	1.996 (0.1189)	1.874 (0.212)	**1.187 (0.146)** ^b^	**1.234 (0.233)** ^b^	1.974 (0.292)
Curved	2.062 (0.187)	2.002 (0.244)	**1.256 (0.16)** ^b^	**1.279 (0.282)** ^b^	1.884 (0.224)
Right					
meanRMS (mv)					
Flat	0.9639 (0.0118)	**3.884 (2.37)** ^b^	1.112 (0.307)	1.237 (0.501)	**3.963 (2.84)** ^b^
Curved	0.9631 (0.0102)	**2.913 (2.07)** ^b^	1.042 (0.406)	1.135 (0.444)	**4.143 (2.73)** ^b^
WFL (nu)					
Flat	4642 (718)	**21,750 (14,300)** ^b^	5822 (2470)	5779 (1930)	**22,720 (17,200)** ^b^
Curved	4400 (718)	**16,180 (12,700)** ^b^	4708 (1760)	5184 (2140)	**22,430 (15,500)** ^b^
SpecDef (Ω/Ω)					
Flat	1.773 (0.1295)	**1.019 (0.227)** ^b^	1.6 (0.334)	1.683 (0.185)	**1.157 (0.432)** ^b^
Curved	1.782 (0.135)	**1.113 (0.277)** ^b^	1.6 (0.273)	1.653 (0.189)	**1.133 (0.337)** ^b^
**Infraspinatus**					
Left					
meanRMS (mv)					
Flat	0.9448 (0.0207)	1.175 (0.309)	**2.158 (1.09)** ^b^	**1.945 (0.762)** ^b^	1.266 (0.433)
Curved	0.9351 (0.0245)	1.194 (0.436)	**1.905 (0.92)** ^b^	**2.168 (1.19)** ^b^	1.064 (0.389)
WFL (nu)					
Flat	6128 (907)	7298 (1860)	**14,390 (6570)** ^b^	**14,910 (9120)** ^b^	8023 (2750)
Curved	5673 (1080)	7178 (2580)	**12,350 (5930)** ^b^	**15,180 (9300)** ^b^	6734 (2570)
SpecDef (Ω/Ω)					
Flat	2.226 (0.266)	2.064 (0.275)	**1.539 (0.305)** ^b^	**1.589 (0.355)** ^b^	1.95 (0.364)
Curved	2.236 (0.283)	2.046 (0.311)	**1.644 (0.32)** ^b^	**1.515 (0.223)** ^b^	**1.916 (0.2)** ^b^
Right					
meanRMS (mv)					
Flat	0.9606 (0.0113)	**1.518 (0.605)** ^b^	1.197 (0.319)	**1.275 (0.361)** ^b^	**1.698 (0.73)** ^b^
Curved	0.9533 (0.0156)	1.239 (0.492)	1.228 (0.551)	0.9943 (0.237)	1.329 (0.596)
WFL (nu)					
Flat	6556 (1230)	**10,040 (4130)** ^b^	7817 (2430)	7998 (2470)	**11,500 (5040)** ^b^
Curved	5887 (1340)	9090 (4820)	7802 (3780)	7122 (3340)	9552 (5130)
SpecDef (Ω/Ω)					
Flat	2.174 (0.217)	**1.831 (0.272)** ^b^	2.07 (0.253)	2.003 (0.282)	**1.731 (0.344)** ^b^
Curved	2.135 (0.229)	**1.803 (0.352)** ^b^	**1.897 (0.279)** ^b^	1.957 (0.246)	**1.798 (0.361)** ^b^
**Teres major**					
** **Left					
stdRMS (mv)					
Flat	1 (7.3e-16)	1.155 (0.249)	**1.703 (0.679)** ^b^	**1.843 (0.796)** ^b^	1.181 (0.366)
Curved	1 (6.89e-16)	1.077 (0.254)	1.58 (0.797)	**1.753 (0.934)** ^b^	1.118 (0.482)
WFL (nu)					
Flat	6296 (1360)	6793 (1880)	**10,070 (3550)** ^b^	**11,010 (5020)** ^b^	6921 (2110)
Curved	5627 (1500)	6267 (2050)	8999 (4470)	9262 (4470)	6239 (2640)
SpecDef (Ω/Ω)					
Flat	2.344 (0.267)	2.199 (0.361)	**1.842 (0.3)** ^b^	**1.851 (0.36)** ^b^	2.131 (0.414)
Curved	2.335 (0.33)	2.17 (0.326)	**1.948 (0.349)** ^b^	**1.87 (0.317)** ^b^	2.04 (0.409)
Right					
stdRMS (mv)					
Flat	1 (7.3e-16)	**1.5 (0.637)** ^b^	**1.362 (0.466)** ^b^	**1.445 (0.461)** ^b^	**1.896 (1.15)** ^b^
Curved	1 (9.21e-16)	1.703 (0.986)	1.187 (0.376)	1.248 (0.52)	**1.824 (0.887)** ^b^
WFL (nu)					
Flat	6371 (1110)	8793 (3430)	8071 (2920)	8358 (2730)	10280 (5330)
Curved	5959 (1280)	8853 (4580)	7662 (3450)	7051 (3130)	9616 (5260)
SpecDef (Ω/Ω)					
Flat	2.043 (0.152)	**1.805 (0.264)** ^b^	1.84 (0.257)	1.92 (0.185)	**1.746 (0.336)** ^b^
Curved	2.032 (0.228)	**1.829 (0.202)** ^b^	1.773 (0.268)^b^	1.765 (0.328)	1.765 (0.352)

a meanRMS: mean of root mean square.

b Bolding indicates significant difference between compressive state and respective control (C2 for flat paddle compressions, C3 for curved paddle compressions) with *P<*.05.

c stdRMS: standard deviation of the root mean square.

d ENT: spectral entropy.

e WFL: waveform length.

f SpecDef: spectral deformation.

g Ω/Ω: Ohm ratio—unitless.

For the sternocleidomastoid, deltoid, infraspinatus, and teres major muscle groups, significant differences were observed only during compression of the ipsilateral breast (the breast on the same side as the muscle being measured). This pattern is demonstrated by the sensitivity clustering in the LCC and LMLO columns for left-sided muscles and in the RCC and RMLO columns for right-sided muscles.

Curved and flat paddle compressions resulted in similar changes in the metric performance. There was no significant difference found between compressions of the 2 paddle designs. This can be similarly extrapolated from Table 3 where pairs of rows generally follow the same trend. Parentheses indicate the larger of the 2 mean values for a given metric and compression. It is important to note that in the case of metrics meanFreq, medianFreq, and DP, a decrease in mean was found in response to the provided stimuli as is commonly expected in physiological response to stressors [23].

**Figure 4.**
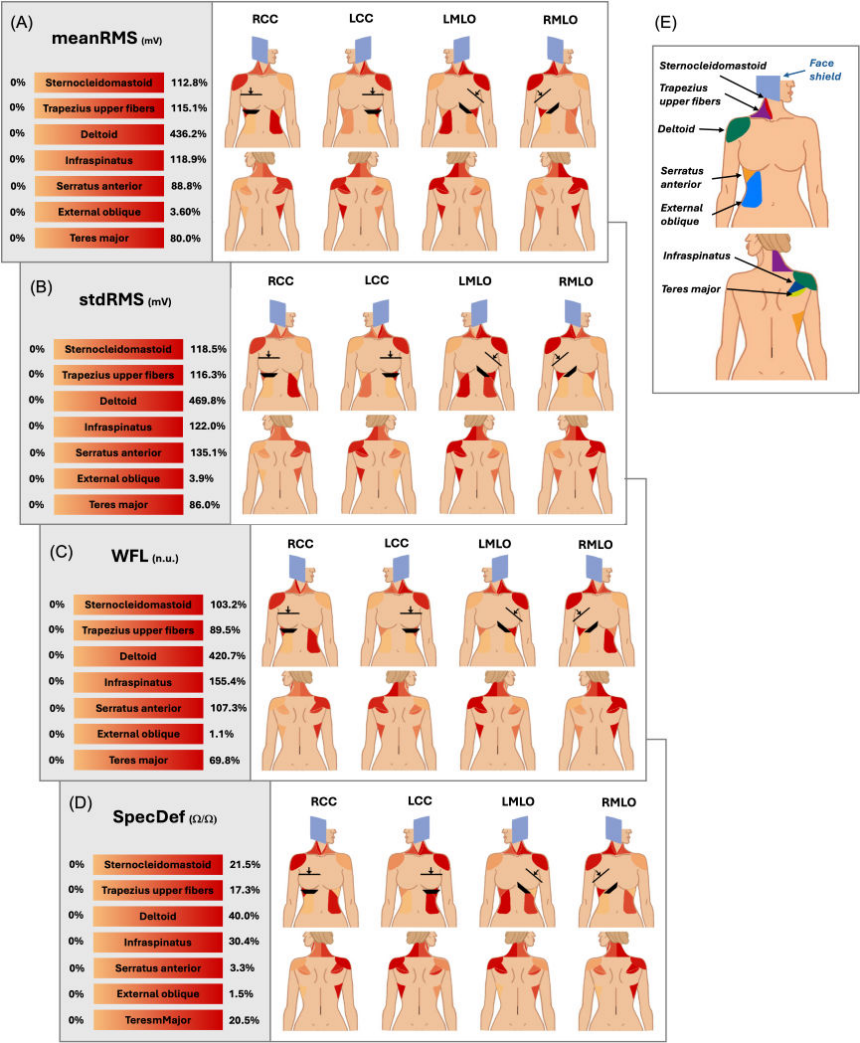
Anatomical heatmap marking absolute percentage change of metrics of interest (A, stdRMS; B, stdRMS; C, WFL; and D, SpecDef) for muscle groups (E) between control and a given compression as participants are positioned against the machine. Curved and flat compression types combined to wholistically represent the 4 mammogram positions. Max% of color bar indicated maximum percentage change considering both left and right components of muscle group. LCC: left craniocaudal; LMLO: left mediolateral; meanRMS: mean of the root mean square; RCC: right craniocaudal; RMLO: right mediolateral; stdRMS: standard deviation of the root mean square; SpecDef: spectral deformation; WFL: waveform length.

Figure 4 clarifies the differences by presenting the absolute percentage change relative to the control for the 4 most responsive metrics of the study. Figure 4E provides a summary key of the muscles of interest. Combining the curved and flat compressions into 1 population (RCC, LCC, LMLO, and RMLO), the physiological heatmaps illustrate the change for all the muscles studied. The deltoid had a maximum percentage change of 436.2% when considering meanRMS (Figure 4A and C). The increase is corroborated by the results in Table 3. Similar percentages are seen across stdRMS, WFL, and SpecDef, noting 469.8%‐420.7% and 40.0%, respectively (Figure 4B-D). The teres major showed a maximum increase of 80.0%, 86.0%, 69.8%, and 20.5% for each of the metrics, which are also significant increases. On average, the sternocleidomastoid increased 112.8%, 118.5%, 103.2%, and 155.4%; the trapezius upper fibers 115.1%, 116.3%, 89.5%, and 17.3%; and infraspinatus 118.9%, 122%, 155.4%, and 30.4% for meanRMS, stdRMS, WFL, and SpecDef, respectively. For the external oblique, averages were 3.6%, 3.9%, 1.1%, and 1.5% for meanRMS, stdRMS, WFL, and SpecDef, respectively.

Before starting the study, participants were asked to report VAS pain ratings for their previous mammogram experiences. The results were between 1 and 9 and had a mean of 5.6 and an SD of 2.1. They again reported a VAS pain rating after finishing the study with respect to the simulated procedure. The results were between 1 and 5 and had a mean of 2.2 and an SD of 1.2. In the second report, participants were also requested to indicate which locations were the most uncomfortable during the study, if any. As indicated in Table 4, the breast was the most often reported location for discomfort, followed by the shoulder and the neck. These reports are summarized in Table 4.

**Table 4.** Reported VAS^a^ pain ratings^b^.

Location	Number of reports
Breast	10
Shoulder	7
Neck	2
Leg	3
Feet	2
None	1
Overall VAS pain reported, mean (SD)	2.2 (1.2)

a VAS: visual analog scale.

b Study sample size (N=25).

Using the same standards but varying the segments, we used statistical comparisons to explore the effects of compression angle as previously discussed. These comparisons demonstrated no notable trends in significance. Similarly, no significance was observed for any metric between the large and small room controls.

## Discussion

This study proposed a multimetric analysis of sEMG activity across 14 different muscles as a methodology to analyze women’s muscular response during a simulated mammogram. The results allowed for a more complete understanding of the machine-patient dynamics and have the potential to further clarify the manifestation of pain or stress relevant to the procedure. To date, this is one of the most complete and thorough studies of EMG activity during mammogram screening.

To arrive at a nuanced understanding of the muscle’s response, 10 EMG-based metrics were used to characterize the signal activity. When compared with related studies, the additional metrics allowed for greater resolution in classifying muscle responses. Out of the 10 metrics, the following 6 frequently indicated differences between the relaxed and stimulated states: meanRMS, stdRMS, medianFreq, meanFreq, WFL, and SpecDef. Out of those 6, meanRMS, stdRMS, medianFreq, and SpecDef showed large differences from one another, with each capturing a distinct characteristic of the signal behavior. In showing greater activation among muscles, these metrics can be reliably used to quantify muscle response throughout the procedure. This reliability is similarly represented in related studies in which greater activation of these metrics was related to greater pain and stress [16,23,24]. Uchiyama et al [20] implemented integrated electromyography, which is a metric that is closely related to meanRMS, and demonstrated similar muscle reactivity when quantifying trapezius, bicep, sternocleidomastoid, and gastrocnemius activation. No prior studies have examined changes in muscle activity trends during mammograms using sEMG recordings and displayed the trends of these 10 metrics. It is important to note that the overall magnitude of percentage change is relative to each muscle so that some muscles may have smaller variations but still represent significant activation.

Analysis of these metrics provides detailed insights into the demanding and stressful positioning required by mammograms. Specifically, the high sensitivity among many of the metrics in the deltoid, teres major, and infraspinatus can be attributed to the strained torsion of the upper torso. Although there is activation relative to the control, the absence of true significance in the external obliques further illustrates this point. The lower torso, including the obliques and abdomen, would normally twist with the upper body to relieve such strain, but mammogram positioning restricts waist and trunk torsion, inducing an uncomfortable, constricted hold. Similarly, in keeping the chest parallel with the imaging table, the patient extends the arm corresponding to the breast being imaged. This position further engages the shoulder or the upper body region and increases the overall demand on the static muscles during imaging. When compared with their baseline, the dominant activation of the deltoid muscles indicates its role as a positioning muscle and a likely source of pain, stress, or fatigue in women during breast compressions. The combined activation of the trapezius upper fibers and the deltoid provides insights into the patient’s lower neck rotation and arm positioning due to the interaction with the face shield. The meanRMS and stdRMS of the associated neck muscles corresponding to the breast being compressed indicated differences for most of the respective compressions. This can be attributed to the engagement of the patient’s arm with the side of the machine as well as the twisting of the head to accommodate the obtrusive face shield.

While visual observation of the body can begin to qualify the described positioning, the complete sEMG analysis illustrates the intensity of the strain that the procedure induces. There is a significant contrast between the involved muscles of the upper extremity and the limited activity of torso muscles. The torso muscles showed no interactions supposed by the imaging table pressing on the breastbone and rib. The data also corroborate the pain locations reported by participants who noted that, after the breast, the shoulders (deltoid, infraspinatus, and teres major) were the most uncomfortable body region during compressions.

A multimetric approach may lead to a more sensitive model that can quantify complex responses involving multiple muscle contributors of varied sizes and locations. This can be seen when comparing the responses of the trapezius upper fiber, deltoid, infraspinatus, and teres major muscle groups. The meanRMS and stdRMS show high activation among all 4 groups (8 muscles). However, out of these, only the deltoid and the infraspinatus muscle groups (4 muscles) show moderate activation for other metrics including slope sign change count, zero crossing count, meanFreq, medianFreq, and DP. This finding could be indicative of a more intense response on behalf of these muscles in comparison with the others. Similarly, it is possible that the additional metrics capture characteristics unique to each muscle that would otherwise be lost if only meanRMS was observed (one of the most common sEMG metrics applied in practice). The study results illustrated this likelihood in a few cases. One such case considers the mean and median frequency response, metrics that are frequently implemented as measures of fatigue [23]. In the results presented, we see cases in which a significant change of these metrics occurs in the deltoid and the infraspinatus but not in the other muscles. This may indicate that the right and left deltoid and the right and left infraspinatus are exposed to more fatigue-inducing strain arising from the type of positioning, repetitive muscle involvement, or some other factor intrinsic to the procedure.

The robustness and sensitivity of the proposed measurement methodology are further strengthened by evidence that they can distinguish lateral variance in compressions. Figure 3 reveals that the metrics that frequently show sensitivity only do so in roughly half of the total compressions. This lateral bias originates from the idea that the activation of a muscle depends on which breast is being imaged. The breast being imaged in turn dictates the location of the muscular tension in the upper torso and neck muscles, isolating the strain to one side in this group of muscles. For example, in the case of the LMLO, the 5 muscle pairs detected a difference more frequently for left breast compressions (flat paddle 8/8 instances, curved paddle 5/8 instances), whereas the RMLO noted a difference mostly for right breast compressions (flat paddle 6/8 instances, curved paddle 5/8 instances) as seen in Table 3. Similar lateral patterns were observed by Uchiyama et al [20]. While these patterns support the sensitivity of the measurement approach, the ability to detect more subtle differences such as those between paddle types may be limited. A larger sample size would likely be required to resolve smaller effect sizes that distinguish paddle-related influences.

While the study relied on a single overall VAS score, which provided meaningful context by identifying common regions of discomfort consistent with our sEMG findings, this format limited direct statistical correlation. Future studies incorporating VAS ratings after each compression would allow a more precise linkage between subjective and objective indicators for complete biomarker development. It is important to note that the reported pain score during the simulated mammogram was lower than the score the participants assigned to their previous experiences. Human ability to recall pain is surprisingly accurate [25]; therefore, the difference in pain is largely attributed to the reduced compressive force used in the study and is not a result of reporting inaccuracies. Limited but sufficient force was applied during the study so that a constant force reading on the machine was achieved. The participant was in a proper, realistic postural positioning without inducing unnecessary compression. As the study did not take any image of the breast, the compressive force applied did not reach the intensities patients normally experience. The standard force ranges from 100 N to 140 N [26], whereas the forces implemented in the study range from 18.25 N to 71.60 N. It is likely that had our study applied the same degree of pressure required by common procedures, the recorded pain metrics would have been more comparable. It can be realistically hypothesized that the painful stimulus of complete breast compression would likely induce additional muscle tension and a further elevated internal stress response. Consequently, the proposed methodology may detect an even greater biomechanical response via EMG mapping. The procedure also maintained the same order of compressions, flat paddle followed by curved paddle, which may have introduced a procedural bias. Given the back-to-back nature of the compressions, the later compressions could have captured patterns corresponding to extended screening, including muscle fatigue or learning results. The influence of this effect was hopefully limited by comparing the muscle activation during compression with flat and curved paddles to the initial C2 and intermittent C3, respectively.

While the proposed methodology was not able to distinguish between paddle types or compression angles, it was able to accurately capture the positional demands associated with each of the 4 different compressions. Supplemental research is needed to detect the effects of paddle design variations through EMG. Future studies incorporating muscles more directly involved in angular positioning may better capture biomechanical differences between vertical and angled compressions. Additionally, angle-dependent effects may exist but with smaller effect sizes than currently observed, suggesting that a larger sample may be required to achieve sufficient statistical power to detect these effects. It is possible that advanced machine learning approaches could be applied to determine the effects of paddle type and other subtle machine design variations on patient discomfort. Likewise, further decomposing the measurement windows could facilitate the exploration of muscle fatigue trends not only across compressions, as examined in this protocol, but also within each individual imaging period. Such within-compression analyses could reveal shorter-term fatigue patterns that complement the broader longitudinal trends. These complex and potentially hidden relationships underscore the importance of a methodology that integrates multiple metrics across several muscles.

Future work should also expand the study population to improve external validity. Including participants with a broader range of ages, BMIs, physical health, breast densities, and demographic backgrounds would help clarify how these factors interact with muscular demand and discomfort during compression. This expanded population would help ensure that findings generalize across diverse patient groups.

As long as the routine mammogram remains a painful and stressful experience, it is crucial to understand the physiological toll the procedure has on patients. This study applied a robust, multimetric sEMG analysis across multiple muscles to objectively characterize the physical impacts of the mammogram procedure. While subjective pain reports provide valuable patient perspectives, EMG offers critical advantages by capturing unconscious muscular compensation patterns, precise temporal responses during specific procedure phases, and quantifiable biomechanical data that patients cannot accurately self-report. By identifying the relative simultaneous activation and inactivation of specific muscles, this methodology can highlight the unique, painful demands of the procedure. In the present analysis, the deltoid and infraspinatus muscles emerged as most consistently responsive across the majority of time domain and frequency domain metrics. This finding suggests their strong potential as candidates for sEMG-based biomarkers of procedural strain. Metrics such as RMS, medianFreq, and WFL, in particular, showed robust sensitivity to compression-specific positional demands. These muscle-metric combinations, therefore, represent promising targets for future refinement of objective indicators of discomfort or biomechanical load during mammography. This metric insight, along with other takeaways on sensitive muscles, provides promising results for potentially highlighting a focus point for improvements to the mammogram procedure that would be difficult to isolate through patient feedback alone. By analyzing a wide range of metrics, the methodology can potentially provide objective pain and stress measures such as those developed in similar studies involving the development of electrophysiological biomarkers [27]. The study methodology demonstrated the sensitivity of sEMG to capture mammogram-specific muscular trends during the compressions, revealing human-machine interaction patterns that inform targeted design modifications. These objective physiological data complement subjective feedback by providing quantifiable, reproducible metrics for assessing proposed improvements to the device design and imaging protocols. Future work should confirm reproducibility through larger cohorts, expanded muscle sets, and test-retest reliability. Relating key muscle-metric patterns to patient discomfort will help establish predictive validity and support the development of sEMG-based biomarkers for mammography-induced strain.

Although women may be reluctantly willing to endure mammograms as a necessary evil, there are many good reasons to better understand and improve the procedure. First, there is the potential to enrich the quality of the medical assessment, including both the results and the patient experience. Second, improvements to the patient experience can go a long way in promoting more widespread application of the technology. Furthermore, the comprehensive methodology proposed lays significant groundwork for future research in machine-human dynamics both in and outside of the health care field. With increased human exposure to novel technologies that modify body posture and muscular kinetics, it is crucial to understand the physiological ramifications in order to allow for their safe and effective application.

## Abbreviations

C1: control recording in a large room (1341 square feet) without mammogram machine
C2: control recording in small room (171 square feet) with mammogram machine before the flat paddle compressions
C3: control recording in small room (171 square feet) with mammogram machine after the flat paddle compressions and before the curved paddle compressions
C_: compression with a curved paddle
DP: drop in power
EMG: electromyography
F_: compression with a flat paddle
LCC: left craniocaudal
LMLO: left mediolateral oblique
meanFreq: mean frequency
meanRMS: mean of root mean square
medianFreq: median frequency
RCC: right craniocaudal
RMLO: right mediolateral oblique
RMS: root mean square
sEMG: surface electromyography
SpecDef: spectral deformation
stdRMS: standard deviation of the root mean square
VAS: visual analog scale
WFL: waveform length
